# Incidence of nutritional support complications in patient hospitalized in wards. multicentric study

**Published:** 2012-06-30

**Authors:** Gloria María Agudelo, Nubia Amparo Giraldo, Nora Luz Aguilar, Beatriz Elena Restrepo, Marcela Vanegas, Sandra Alzate, Mónica Martínez, Sonia Patricia Gamboa, Eliana Castaño, Janeth Barbosa, Juliana Román, Ángela María Serna, Gloria Marcela Hoyos

**Affiliations:** a Research Group on Food and Human Nutrition, Universidad de Antioquia, E-mail:gmao@quimbaya.udea.edu.co; b Hospital General de Medellín, E-mail: beatrizrestrepo@une.net.co; c Hospital Pablo Tobón Uribe. E-mail: salzatej@hptu.org.co; d Hospital Universitario San Vicente de Paúl, E-mail: monicamar2@yahoo.es; e Instituto Neurológico de Antioquia, Email: sonygamboa@gmail.com; f IPS Universitaria, E-mail: elikace@yahoo.es; g Clínica Las Américas, E-mail: janebarbosab@gmail.com

**Keywords:** Nutritional support, enteral nutrition, parenteral nutrition, complications, adults, hospitalization

## Abstract

**Introduction:**

Nutritional support generates complications that must be detected and treated on time.

**Objective:**

To estimate the incidence of some complications of nutritional support in patients admitted to general hospital wards who received nutritional support in six high-complexity institutions.

**Methods:**

Prospective, descriptive and multicentric study in patients with nutritional support; the variables studied were medical diagnosis, nutritional condition, nutritional support duration, approach, kind of formula, and eight complications.

**Results:**

A total of 277 patients were evaluated; 83% received enteral nutrition and 17% received parenteral nutrition. Some 69.3% presented risk of malnourishment or severe malnourishment at admittance. About 35.4% of those receiving enteral nutrition and 39.6% of the ones who received parenteral nutrition had complications; no significant difference per support was found (*p*= 0.363). For the enteral nutrition, the most significant complication was the removal of the catheter (14%), followed by diarrhea (8.3%); an association between the duration of the enteral support with diarrhea, constipation and removal of the catheter was found (*p* < 0.05). For parenteral nutrition, hyperglycemia was the complication of highest incidence (22.9%), followed by hypophosphatemia (12.5%); all complications were associated with the duration of the support (*p* < 0.05). Nutritional support was suspended in 24.2% of the patients.

**Conclusions:**

Complications with nutritional support in hospital-ward patients were frequent, with the removal of the catheter and hyperglycemia showing the highest incidence. Duration of the support was the variable that revealed an association with complications. Strict application of protocols could decrease the risk for complications and boost nutritional support benefits.

## Abstract (es)

**Introducción:**

El soporte nutricional genera complicaciones que deben detectarse y tratarse oportunamente.

**Objetivo:**

Estimar la incidencia de algunas complicaciones del soporte nutricional en pacientes hospitalizados en salas generales que recibieron soporte nutricional en seis instituciones de alta complejidad.

**Métodos:**

Estudio multicéntrico, descriptivo, prospectivo en pacientes que recibieron soporte nutricional. Las variables estudiadas fueron diagnóstico médico, estado nutricional, duración del soporte, vía de acceso, tipo de fórmula y ocho complicaciones.

**Resultados:**

Se evaluaron 277 pacientes; 83% recibieron nutrición enteral y 17% parenteral. El 69.3% presentaron al ingreso riesgo de malnutrición o malnutrición severa. El 35.4% que recibieron nutrición enteral y el 39.6 % de los que recibieron parenteral presentaron complicaciones, sin diferencia significativa por tipo de soporte (*p*= 0.363). Para la nutrición enteral, la complicación más incidente fue el retiro de sonda (14%) seguida de la diarrea (8.3%); se encontró asociación de la duración del soporte con la diarrea, estreñimiento y retiro de sonda (*p* < 0.05). Para la nutrición parenteral, la hiperglicemia fue la complicación de mayor incidencia (22.9%) seguida por la hipofosfatemia (12.5%); todas las complicaciones se asociaron con la duración del soporte (*p* < 0.05); en el 24.2% de los pacientes fue suspendido el soporte.

**Conclusiones:**

Las complicaciones del soporte nutricional en pacientes de salas fueron frecuentes, mayor incidencia el retiro de sonda y la hiperglicemia. La duración mostró asociación con las complicaciones. La aplicación estricta de los protocolos podría disminuir el riesgo de las complicaciones y potenciar los beneficios del soporte nutricional.

## Introduction

Sick patients admitted to a hospitalization ward may compromise their nutritional state because of increased nutrient and energy requirement, decreased food intake, or both. By norm, the oral route is the election to provide the nutritional and energy requirements to a hospitalized patient; however, there are situations in patients do not want, cannot, or should not use this route. In these cases, nutritional support (NS) represents the only feeding option to avoid deterioration of the nutritional state and contribute to recovery^1^. In hospitalized patients, enteral nutrition (EN) is used between 33% and 92% of the cases and parenteral nutrition (PN) is used between 12% and 71% of the cases^2^. Nutritional support has proven benefits on healing processes, catabolic response, decreased incidence of complications and hospital stay, which is reflected in the diminished morbidity and mortality of these patients^3^. 

Currently, equipment, techniques, products, and guides are available to permit offering patients safe and efficient NS; however, during its administration both EN as PN can present mechanical, metabolic, gastrointestinal, and infectious complications. Lamache *et al*., estimate that between 10 and 15% of patients receiving EN may register some type of complication, of which from 1 to 2% can be serious^4^. Regarding PN, hepatobiliary complications are reported between 15 and 85% of the patients^5^, 9.3% related to access of the catheter^6^ and up to 56.5% to metabolic complications^6^ ^-^ ^7^ The systematic studies reporting the incidence of NS complications in patients hospitalized in wards are few; no data are published for Colombia.

### Objective

This study estimated the incidence of certain complications of nutritional support in patients hospitalized in general wards in high-complexity institutions.

##  Materials and Methods 

This was a multicentric, descriptive, prospective study carried out in six high-complexity institutions in the city of Medellín (Colombia) during a four-month period. The target population was constituted by adult patients over 18 years of age who were admitted to general wards and received NS for at least 48 hours. Gestational women and patients remitted from other hospital units or from other institutions with nutritional support established were not included in the study. In each participating institution, once patients fulfilling the inclusion criteria were selected, the presence of the complications defined for each type of NS was registered; the complication was reported only once as a new event.

Before starting the study, each participating institution provided general information on the institution, research nutritionist responsible for gathering information and statistics of patients receiving NS. The group of researchers held preliminary meetings to agree on the complications to be studied, the criteria to define them, and unify the collection of information. To control bias, the researchers received training to identify and register the different complications according to that previously established and the filling out of forms was standardized. Meetings were held each month to solve issues and review the consolidated figures submitted.

The following information was registered from each patient selected: age, gender, medical diagnosis, nutritional state upon admission via subjective global assessment (SGA), type of nutritional support, access route, type of formula, complications, duration of support, suspension and cause. A pilot test was conducted during a month that permitted testing and adjusting the process to gather information for the study.

### Definition of complications: 

according to reports from other studies, the protocols implemented in the participating institutions and the clinical experience of the researchers, the following complications per type of NS were defined.

#### Enteral nutritional support (ENS) 

##### High gastric residual (HGR):

residual with nutritional characteristics greater than 150 mL. Diarrhea: more than five stool movements of liquid consistency during a 24-hour period or two stools with a volume above 1,000 cc/day. Constipation: patient who does not have a stool movement every three days once EN is started. Catheter removal (CR): voluntary and involuntary extraction of the catheter. Removals per medical order were excluded.

#### Parenteral nutritional support (PNS)

##### Hyperglycemia (HG):

if for every three values obtained during the day, at least two of these were greater than 150 mg/dL. Cholestasis: in patients with more than two weeks with parenteral support and who present at least one of the following alterations: total or direct bilirubin > 1.2 mg%, alkaline phosphatase >280-380 IU/L, gamma glutamyl transferase >50 IU/L. Sepsis associated to catheter: identification of the same microorganism in a blood culture and from a part of the catheter, culture done semi-quantitatively or quantitatively in the presence of signs of infection barring other causes. Hypophosphatemia (HP): values of blood phosphorus below 2.5 mg/dL.

### Ethical Management of the Investigation:

The investigation was classified with minimum risk according to the Colombian Ministry of Social Protection in resolution number 008430 of October, 1993 article 11; all the ethical principles were complied for medical research on human beings according to the Helsinki Declaration from the World Medical Association. All patients signed an informed consent and the research was approved by three Bioethics Committees.

### Statistical analysis:

the data base and the statistical analysis of the information were performed in the SPSS program version 18.00. The quantitative variables were described via measures of central tendency and dispersion; the qualitative variables were measured via frequencies and percentages. For each complication, incidence and density of incidence (Nº episodes*100/days of NS) were calculated. The Chi^2^ test or Fisher's exact test were used to explore the association of each complication according to the variables of interest and the Mann Whitney U when at least one of the variables was quantitative. The level of significance defined was *p* < 0.05.

## Results

The final population was comprised of 277 patients of which 134 (48.4%) were males. The mean age was 63.4 ± 19.3 years and 85% were over 40 years of age. Of the total number of patients, 159 (57.4%) were admitted per medical diagnosis (cardiovascular, gastrointestinal, neurological, respiratory, renal, hepatic disease, and human immune-deficiency virus), 43 (15.5%) per neoplasia, and 41 (14.8%) per surgery. Some 69.3% presented upon admission risk of malnutrition or severe malnutrition. Table 1 describes the general characteristics of the study population per type of support. No association was found between the type of NS and the medical diagnosis upon admission (*p*= 0.058), nor with the nutritional diagnosis per SGA (*p*= 0.316).

Regarding the type of NS, 82.7% (229) of the patients received EN and 17.3 % (48) received PN. The access route most used for EN was the nasogastric tube (57.2%) and the subclavian vein for PN (89.7%); the polymer formula was the most used in EN (51.5%). The duration of the support was on the average 12 ± 12 days, with a minimum of two and a maximum of 79 days; and in 59.8% of the cases, the duration was less than 10 days without significant difference per type of support. The total of days for EN was 2,793 days and PN it was 652 days. Table 2 describes the characteristics per type of NS.

Of the patients studied, 35.4% of those receiving ENS and 39.6% of those receiving PNS presented complications, without significant difference per type of support (*p*= 0.363).Catheter removal was the complication of greatest incidence in patients receiving EN (14.0%) with a density of incidence of 0.50 episodes per 100 days of support. In PNS, the complication of greatest incidence was HG (22.9%) with a density of incidence of 3.51 episodes per 100 days of support. In general, 1.27 complications occurred per every 100 days for EN and 6.01 complications for PN.

**Table 1:**
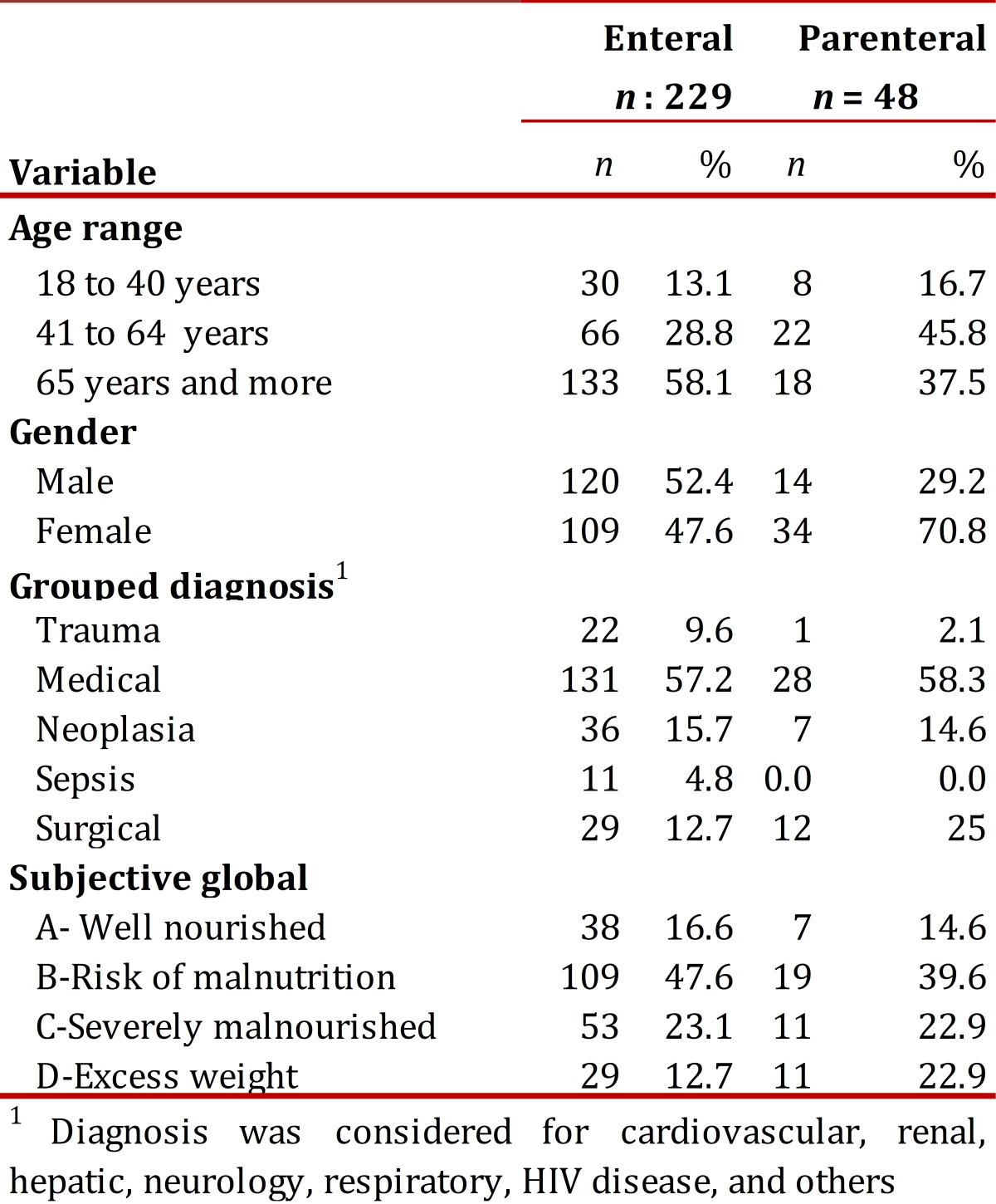
General characteristics of the study population

**Table 2.**
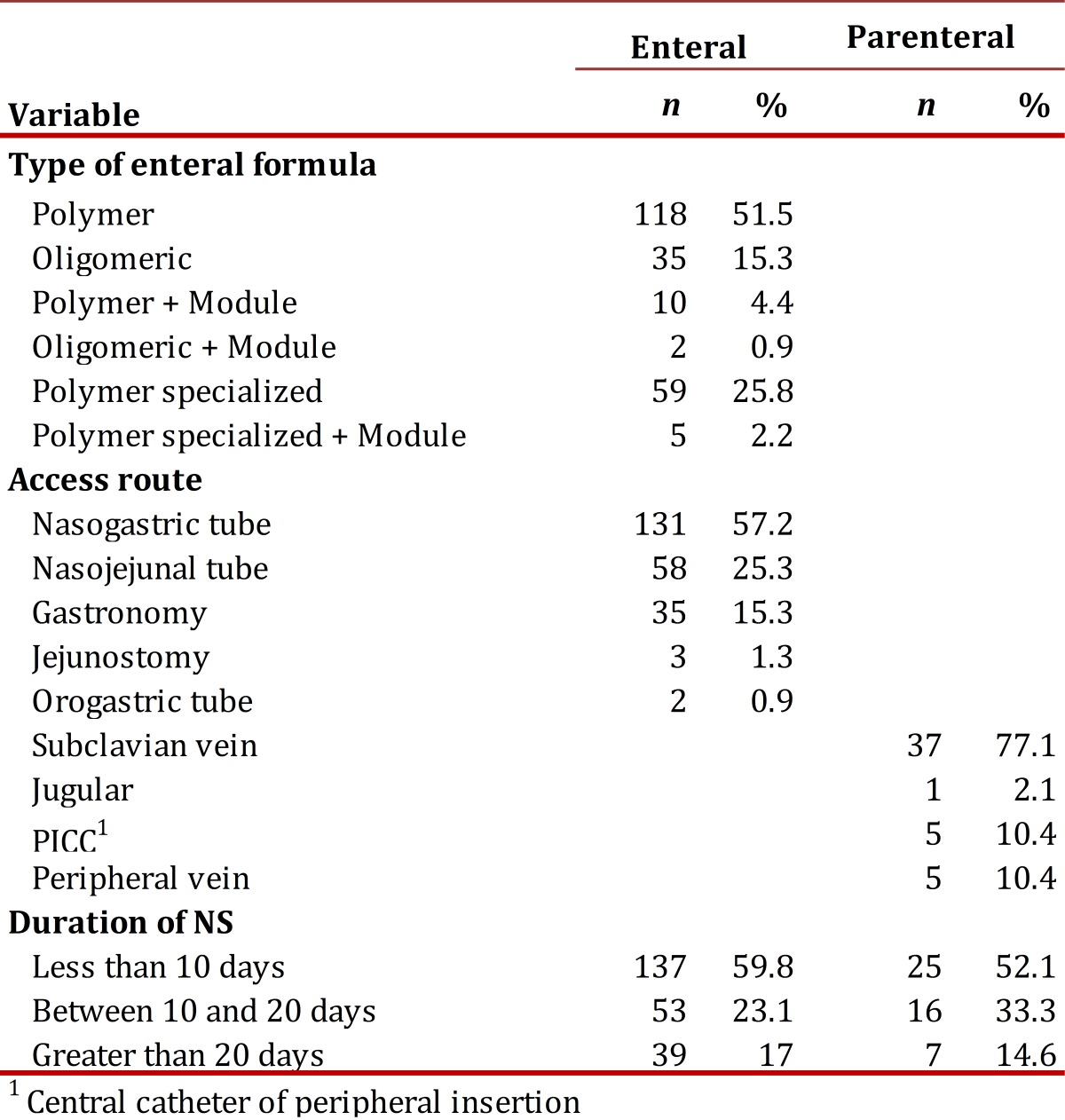
Characteristics of the nutritional support of the study population

Tables 3 and 4 present the incidence and density of incidence of complications per type of NS. The duration of enteral and parenteral NS was the only variable that showed significant association with most of the complications studied (Tables 5 and 6). The NS was suspended in 24.2% of the cases and the main reasons were death (61.2%) and catheter removal (20.9%).

**Table 3:**
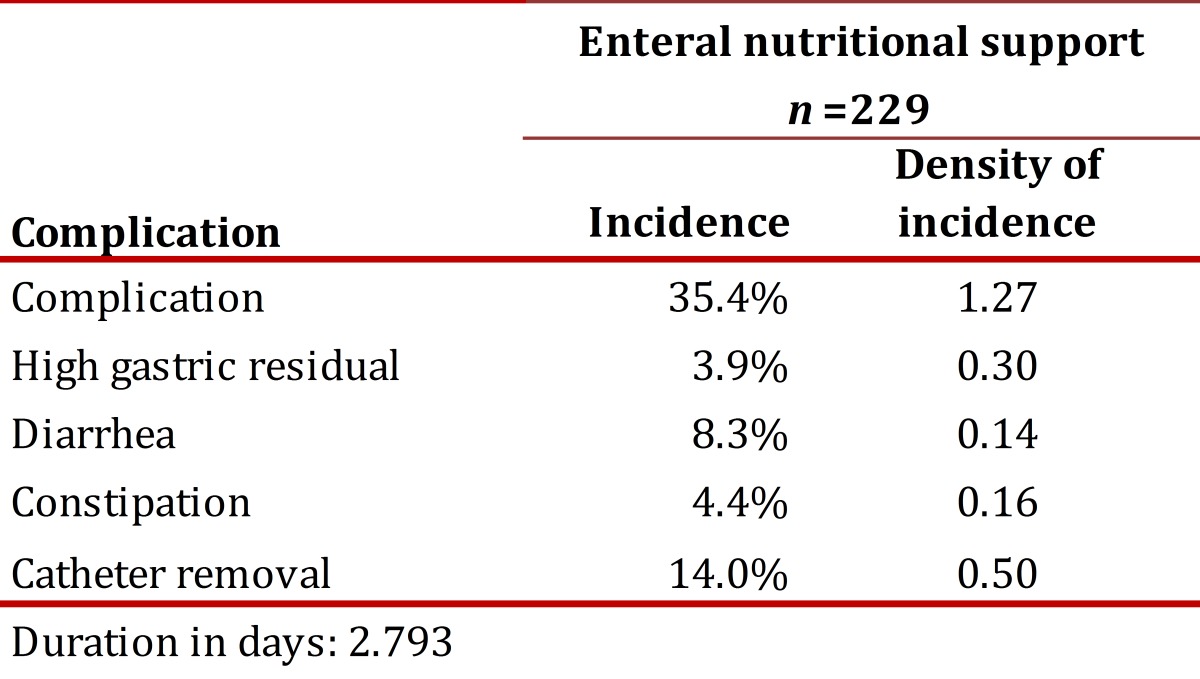
Incidence and density of incidence of the complications studied of enteral nutritional support

**Table 4.**
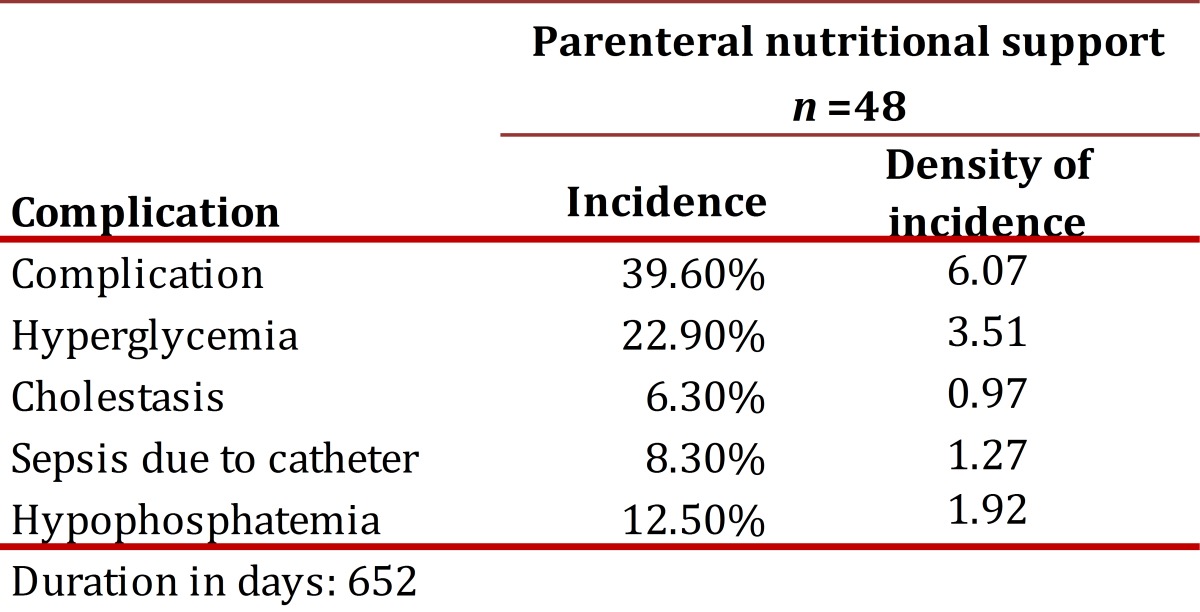
Incidence and density of incidence of the complications studied of parenteral nutritional support

**Table 5:**
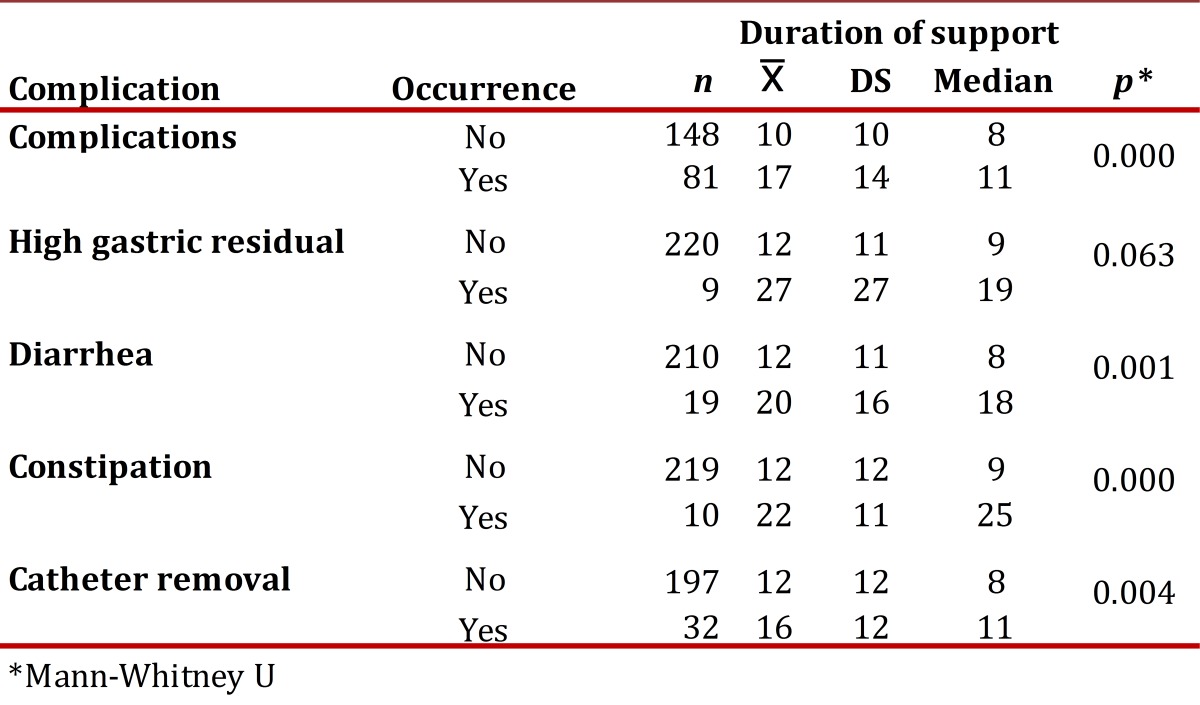
Complications of enteral nutritional support according to duration

**Table 6:**
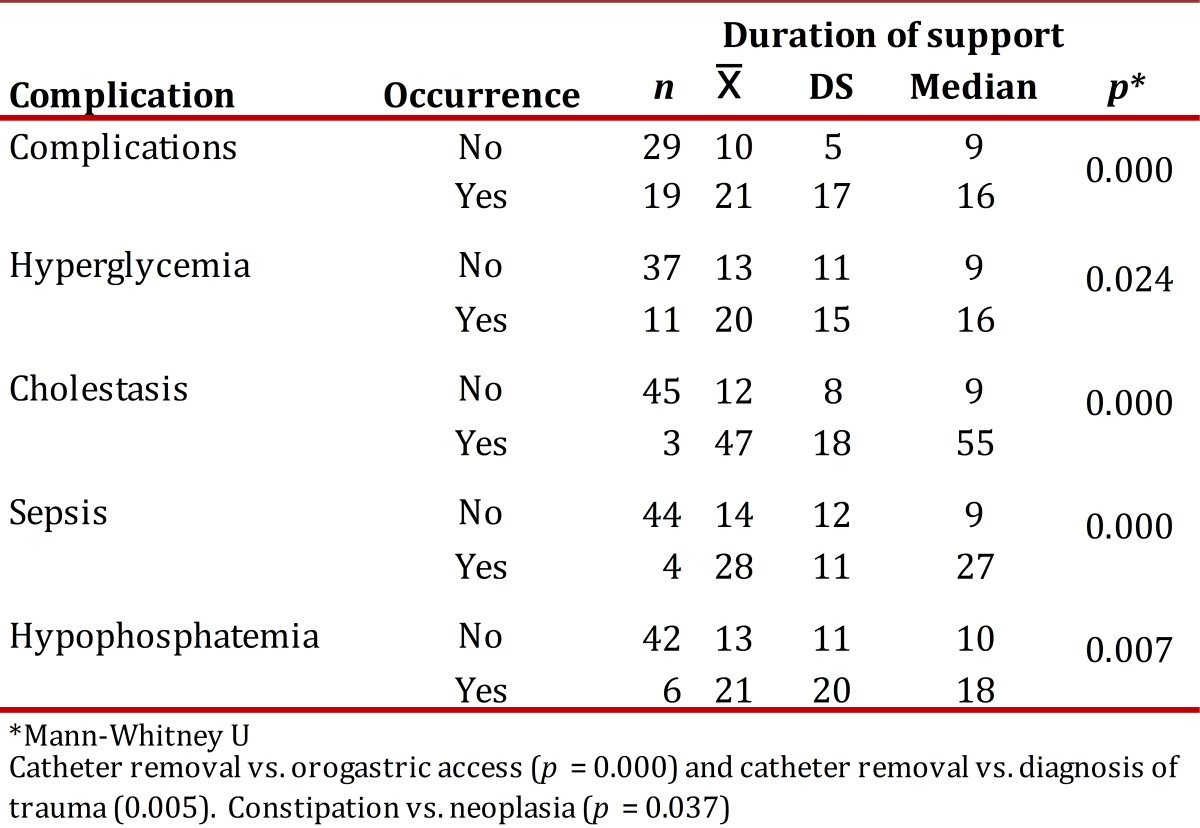
Complications of parenteral nutritional support according to duration

## Discussion

Nutritional support is an alternative in providing the energy and nutrients required by hospitalized patients when because of some conditions it is not possible to feed via oral route. Although ENS- because it is more physiological - has shown lower risk of complications than PNS, the results in this study did not reveal significant differences in the incidence of the complications per type of support (*p*= 0.363).

The complication of EN of greatest incidence was voluntary or involuntary catheter removal. The incidence was of 14% with 0.50 episodes per every 100 days of support. Although it is a frequent situation, no data have been published on the incidence of this complication for comparative purposes. However, it has been reported that it is more common in children, neurological patients, and in those receiving sedatives. Catheter removal may be a factor compromising the nutritional contribution by the temporary or permanent suspension of the support. In this study, this was the most frequent cause of ENS suspension. On the other hand, reinserting the catheter may cause traumatism and complications to the patient; on some occasions it may be necessary to use another access and under exceptional cases change PNS. Given that it is a frequent complication and that it bears consequences on the nutritional condition of patients receiving ENS, it is important to implement measures that can prevent it like permanent monitoring and use of nasal fixings currently available in the market.

Diarrhea was the second complication of incidence of EN, with 8.3% and 0.14 events per every 100 days of support. The etiology of diarrhea may be related with the type of formula and the technique of administration, as well as with the base disease, the presence of infections, and some medications administered (antibiotics, laxatives, antacids, prokinetic, antiarrhythmic, cytotoxic, immunosuppressive).The consequences of this complication are associated to loss of liquids and electrolytes, malabsorption of nutrients, and infections on occurrence of ulcers by pressure^8^. Frequently, EN is considered one of the main causes of diarrhea, but the factors previously cited must be considered. Regarding this, Edes *et al*., evaluated the causes of diarrhea in patients fed via catheter and found that in only 21% of the cases was it attributed to EN, while medications were directly responsible in 61%, and infection due to *Clostridium difficile *in 17% of the cases^9^. Because of this, when there is diarrhea, it is important to evaluate other probable causes before making the decision to suspend ENS. The results of this study are not comparable to other studies, given the lack of uniformity in its definition and the figures vary between 20.7% and 72.4%; however, when applying objective definitions - as in this work - the incidence diminishes to values between 10% and 18%^8^.

The incidence of constipation was of 4.4% and 0.16 events per every 100 days of ENS. Although it has not been widely studied as a complication, figures up to 15.7% have been reported. Among other causes, this complication is attributed to low contribution of liquids, an insufficient amount of fiber, immobilization, and to the use of medications like anticholinergics and opiates^10^. However, few controlled studies have contributed to clarify the etiology of constipation in patients receiving EN and it is speculated that besides the prior factors, alterations may be involved in intestinal motility as a consequence of the pathological process presented by patients^11^. For its treatment, fiber plays a determinant role, especially in those receiving prolonged EN. Although data are not available to compare the findings with respect to this complication, it is considered that its incidence was low, possibly because they were non-critical patients, with some degree of mobility and lower possibility of medications that interfere with intestinal motility.

High gastric residual was the complication of lowest incidence (3.9% with 0.30 events per every 100 days of ENS).Values reported vary between 20 and 70%, although most data come from studies of patients in critical state^12^. Particularly, the multicentric study from which this study is derived, evaluated the complications of NS in patients in Intensive Care Units, finding HGR as the complication of greatest incidence (24.9%)^13^.These differences found per hospitalization unit may be because critical patients can present special conditions given by the severity of the diagnosis, uroendocrine alterations, gastric emptying alterations, hemodynamic compromise, hypermetabolic states, and the collateral effects of prescribed medications like opiates and inotropics. As part of the follow up of tolerance to ENS, measurement of gastric residual is routine; however, the cut-off points to define it are still controversial and vary from 150 mL to 500 mL. The most recent recommendations suggest higher values and others propose further evaluating the tendency of the residuals during the day and not diagnose it with only one reading^14^.

Regarding complications of PNS, in this study HG had the highest incidence, 22.9% and 3.51 events per every 100 days of PNS. The excess of dextrose in PN has been recognized as the most common cause of HG. In hospitalized patients it is suggested to maintain blood glucose values between 80 and 110 mg/dL and values below 145 mg/dL to reduce morbidity and mortality. Among other consequences, HG alters the immune response by interfering with phagocyte activity, increasing inflammatory response, and inducing apoptosis, which also increases risk of mortality^15^.

Few studies report the incidence of HG in patients with PN in wards. Pleva *et al*., in a retrospective study, reported an incidence of 44%^16^; Ahrens *et al*., in a controlled clinical trial with surgical patients receiving PN, found an incidence of 33% in the group receiving a greater calorie contribution^17^. Marti-Bonmati *et al*., in a multicentric study found a prevalence of 26.7%^18^. The incidence of HG in our study was lower compared to those previously cited, differences that could be explained by the heterogeneity in the cutoff points established, given that while this study defined it as values above 150 mg/dL during the same day, the rest used as cut-off point a value greater than 200 mg/dL.

In this study, HP was the second complication of greatest incidence for PN (12.5%). It is considered a serious electrolytic disorder, given that phosphorus maintains the integrity of the cellular membrane and it is a cofactor of diverse metabolic pathways for energy formation. Few studies report the incidence of HP in patients in wards; Martínez *et al*. reported 18.1% in post-surgical patients^19^ and Llop *et al*., reported 17.7%^20^ - values greater than those found in this study, which could be explained by the fact of having included post-surgical patients in the first and critical patients in the latter, who were under conditions of greater stress making them susceptible to enduring this complication. Hypophosphatemia has been widely associated to the re-feeding syndrome and many studies report its frequency and consequences in patients receiving

PNS. This study shows that the complication is frequent and that more strict surveillance is necessary to detect and prevent it because of its implications among which there are severe muscular, respiratory, and cardiac weakness, and under extreme cases, sudden death^21^.

Sepsis due to catheter has been considered one of the most serious complications of PNS and it is associated to increased costs of care, hospital stay, morbidity and mortality, besides being a frequent cause of NS suspension with the consequences that this may have for patients in their nutritional state. In this study, the incidence of sepsis due to catheter was of 8.3% and the density of incidence of 1.27 events per every 100 days of hospitalization. An incidence between 1.3% and 26.2% has been reported for this complication; the variation in the values may be due to differences in the study designs, definition of the complication, and diversity in populations studied^22^. Although PN has been suggested as an independent risk factor for infection, lower colonization (1.6%) has been reported in catheters used for PN compared to those used for the administration of liquids, medications, or dialysis (12%-21%); none of those used for PN presented infection, results attributed to the application of strict protocols and to the exclusivity of the catheter for PN^23^. Although it has been suggested that this complication is a consequence of non-compliance of the protocols in the insertion and maintenance of the catheter^24^, factors like malnutrition, sustained hyperglycemia, and days of hospitalization prior to insertion of the catheter have been associated to greater risk^23^; immunosuppression associated to malnutrition, microbial contamination/colonization of the catheter and of the skin around the insertion site have also been identified as causes of this complication. This study found significant association between risk of sepsis due to catheter and greater duration of NS.

Hepatic steatosis is a frequent complication of PN, which is associated to long periods of this type of support. Parenteral nutrition is considered an absolute risk factor for the formation of biliary sludge and gallstones. Its etiology is multifactorial, suggesting among other factors, excess of calorie and lipid contribution, nutrient deficiencies, sepsis, and lack of enteral stimulation. The incidence of hepatic complications in adults have been reported in broad ranges, from 20 to 75% and seem less severe and frequent in patients receiving oral feeding^25^.The incidence of cholestasis in this study was of 6.3% and the density of incidence of 0.97. The higher duration of the nutritional support was significantly associated to risk of hepatic steatosis; hence, in patients provided with prolonged PNS, all the measures tending to diminish its incidence must be implemented, among others, it is recommended to avoid energy excess, provide infusions with a balanced composition of nutrients, cycle PN, avoid sepsis, and start EN as soon as possible.

In conclusion, NS poses risks and the gastrointestinal, mechanical, infectious, and metabolic complications may emerge in patients receiving both EN as well as PN. Most studies report the incidence of these complications in patients in Intensive Care Units, but few mention patients in wards. This study found catheter removal as the complication of greater incidence of ENS and HG is the most frequent complication of PNS. Strict application of NS protocols and their implementation by trained teams may contribute to diminishing the risk of complications so that such will finally accomplish its purpose in candidate patients to receive it.
